# Benign Convulsions with Mild Rotavirus and Norovirus Gastroenteritis: Nationwide Data from the Health Insurance Review and Assessment Service in South Korea

**DOI:** 10.3390/children8040263

**Published:** 2021-03-30

**Authors:** Dong Hyun Kim, Dong Jun Ha, Yeong Seok Lee, Min Jun Chun, Young Se Kwon

**Affiliations:** Department of Pediatrics, Inha University School of Medicine, Incheon 22332, Korea; id@inha.ac.kr (D.H.K.); djha0917@inhauh.com (D.J.H.); yslee1016@inhauh.com (Y.S.L.); mjchun0716@inhauh.com (M.J.C.)

**Keywords:** rotavirus infection, norovirus infection, benign convulsions with mild gastroenteritis

## Abstract

There have been no large-scale studies on the epidemiology of benign convulsions with mild gastroenteritis (CwG) since the introduction of the rotavirus vaccine in South Korea in 2007. This study aimed to analyze the trends in rotavirus gastroenteritis (RVGE) and rotavirus-associated CwG (RaCwG) after rotavirus vaccination. Further, we aimed to analyze changes in norovirus gastroenteritis (NVGE) and norovirus-associated CwG (NaCwG) using nationwide data from the Korean Health Insurance Review and Assessment Service. Between 2007 and 2019, this study analyzed children aged <6 years who were diagnosed with RVGE, NVGE, RaCwG and NaCwG. The changes in the prevalence of each disease and the ratio of CwG to enteritis were analyzed and the effects of age, sex and season were also analyzed. RVGE, RaCwG, NVGE and NaCwG were diagnosed in 273,898, 4246, 35,593 and 337 patients, respectively. The prevalence of RVGE was on a decreasing trend every year, but the prevalence of NaCwG and NVGE was on an increasing trend. There was a significant annual increase in the ratio of CwG to enteritis in both viruses. In order to control the prevalence of RaCwG, measures other than the rotavirus vaccine are required and measures to prevent norovirus are necessary.

## 1. Introduction

Annually, more than 100 million individuals present with infectious enteritis with some children requiring hospitalization. Rotavirus and norovirus, which are the major causes of infectious enteritis, are responsible for 520,000 and 200,000 annual deaths, respectively, in children aged <5 years [1,2]. Rotavirus and norovirus are transmitted through the fecal–oral route; moreover, it causes fever or intestinal symptoms, including vomiting and diarrhea [3]. In addition, there are some reports that rotavirus and norovirus can cause neurological disorders, including meningitis, encephalitis and encephalopathy [4,5,6].

Benign convulsions with mild gastroenteritis (CwG) is a neurological disorder caused by an enteric virus and was first described in 1982 by Morooka in children aged 1 month to 6 years without a history of convulsive disease in the absence of fever [7]. Komori reported the following CwG characteristics [8,9]: (1) occurrence of afebrile convulsions within 5 days of acute viral enteritis in a previously healthy child; (2) absence of moderate/severe dehydration; (3) no abnormality in the cerebrospinal fluid test, serum electrolyte level and blood sugar level; (4) good prognosis; and (5) acute illness. Although CwG is often reported in East Asian countries, including South Korea and Japan, it has also been reported in the United States and Europe [10,11,12].

In 2019, the World Health Organization recommended that all countries include rotavirus vaccines in the National Immunization Program [13]. In South Korea, RotaTeq^®^ and Rotarix® were introduced in June 2007 and March 2008, respectively. Subsequently, there has been an annual increase in the rotavirus vaccination rates of South Korean infants born in 2007 (5.1%), 2008 (26.1%), 2011 (45.2%) and 2017 (85.6%) [14].

Currently, there are clinical studies on several norovirus vaccines, including oral monovalent vaccines and bivalent intramuscular vaccines [15,16]. However, there is no commercially available norovirus vaccine.

Since the rotavirus vaccine was introduced, there has been an overall decrease in the prevalence of rotavirus gastroenteritis (RVGE); in contrast, the prevalence of norovirus gastroenteritis (NVGE) has annually increased and has become a major enteric virus [17,18,19,20]. However, there have been limited national epidemiological studies for rotavirus-associated CwG (RaCwG) and norovirus-associated CwG (NaCwG).

This study aimed to use post-vaccination data from the National Health Insurance Review and Assessment Service (HIRA) database to determine changes in the prevalence of RVGE, NVGE, RaCwG and NaCwG, as well as seasonal changes in the ratio of RaCwG to RVGE and NaCwG to NVGE, after the introduction of the rotavirus vaccine.

## 2. Materials and Methods

### 2.1. Data Sources

National Health Insurance covers the entire population of South Korea [21]; consequently, it contains >98% of national medical information [22]. Using the HIRA database, medical researchers can access diagnostic codes and de-identified patient medical records. This study employed the HIRA database to determine the number of patients who visited a clinic or hospital with a diagnosis of RVGE, RaCwG, NVGE and RaCwG. Subsequently, we used the mid-year population (MYP) obtained from the Korean Statistical Information Service (KOSIS) to identify changes in annual prevalence, as well as to compare the prevalence rates over specific periods. This study was approved by the Institutional Review Board of Inha University Hospital (IRB no. 2020-01-036).

### 2.2. Patient Criteria

We obtained medical records of patients aged <6 years between 2007 and 2019. Since data for 2019 were only available until May, we only included the data for 2018.

In South Korea, when diagnosing viral enteritis, a diagnosis code corresponding to the related viral enteritis is usually input when positive in Reverse transcription polymerase chain reaction (RT-PCR) or enzyme linked immunoassay (ELISA). Taking this into account, we considered patients with RVGE and NVGE as those with diagnostic ICD-10 codes of A08.0 and A08.11, respectively. Among these patients, we excluded patients diagnosed with “acute gastroenteropathy” (A08.1), “adenoviral enteritis” (A08.2), “viral gastroenteritis” (A08.30) and “astroviral gastroenteritis” (A08.31).

Among RVGE and NVGE patients, those who met the CwG criteria were classified as RaCwG and NaCwG, respectively; moreover, cases with “convulsion” (R56) and “other and unspecified convulsions” (R56.8) were included. Additionally, for newborns aged <29 days, cases with “convulsion of newborn” (P90) were included. Based on the criteria described by Komori et al. [9], we excluded patients with the following diagnostic codes: (1) “febrile convulsions” (R56.0); (2) “dehydration” (P74, E86); “disorders of fluid, electrolyte and acid-base balance” (E87) or “hypoglycemia” (E16, P70); (3) “meningitis” (A87, G00, G01, G02, G03); “encephalitis” (B00.4, A83, A84, A85, G04, G05); and (4) “epilepsy” (G40).

### 2.3. Analysis of Changes in the Prevalence of RaCwG, RVGE, NaCwG and NVGE

To calculate the annual incidence per 100 individuals of RaCwG, RVGE, NaCwG and NVGE, the number of patients having each disease relative to the MYP from 2007 to 2018 were used. In addition, as the rotavirus vaccination rate increased [14], the trend of annual changes in the incidence of RaCwG and RVGE was identified. In order to analyze this change according to the rotavirus inoculation rate, the period was divided into period A (2007−2012) and period B (2012−2018), as 2012 was five years after the introduction of the rotavirus vaccine in South Korea. The prevalence of rotavirus was compared.

To analyze the effect of additional factors on the prevalence of RaCwG, RVGE, NaCwG and NVGE, the incidence of disease was separated by age, sex and season.

### 2.4. Analysis of the Ratio of CwG to RVGE or NVGE

By calculating the ratio of RaCwG to RVGE and the ratio of NaCwG to NVGE per year, the change in the incidence of CwG patients among enteritis patients was analyzed. To analyze the effect of additional factors on the ratio of RaCwG to RVGE and the ratio of NaCwG to NVGE, we analyzed by age, sex and season.

### 2.5. Statistical Analysis

Data analysis was performed with remote access to the HIRA database using SAS Enterprise version 9.2 (SAS Institute, Cary, NC, USA). Statistical analyses were performed using SPSS version 19.0 (IBM, Armonk, NY, USA). Using negative binomial regression anlaysis, annual prevalence changes of RaCwG, RVGE, NaCwG and NVGE and changes in prevalence according to age, sex and season were analyzed. Furthermore, using negative binomial regression analysis, not only were the annual change of the ratio of RaCwG to RVGE and the ratio of NaCwG to NVGE examined, but also the change according to age, sex and season were analyzed. Prevalence changes in periods A and B were analyzed using chi-square analysis. Statistical significance was set at *p* < 0.05.

## 3. Results

### 3.1. Characteristics of Patients with RVGE, RaCwG, NVGE and NaCwG

Between 2007 and 2018, there were 273,898 and 35,593 patients with RVGE and NVGE, respectively. Among them, 4246 and 337 patients had RaCwG and NaCwG, respectively. Patients with RVGE (37.8%) and RaCwG (63.1%) accounted for the highest proportions in the <1-year-old group. Further, patients with NVGE (30.0%) and NaCwG (59.9%) accounted for the highest proportions in the 1–2-years-old group. Additionally, patients with NVGE (44.5%) and NaCwG (50.4%) accounted for the highest proportions during winter. In the 1–2-years-old group, the mean prevalence of RaCwG and NaCwG per 100 comprising the MYP was 0.013 and 0.00001, respectively.

The ratio of male and female was 54.8% and 45.2% in patients with RVGE, 52.1% and 47.9% in patients with RaCwG, 54.6% and 45.4% in patients with NVGE and 45.4% and 54.6% in patients with NaCwG, respectively (Table 1).

### 3.2. Annual Changes in the Prevalence of RaCwG, RVGE, NaCwG and NVGE

**Figure 1:**
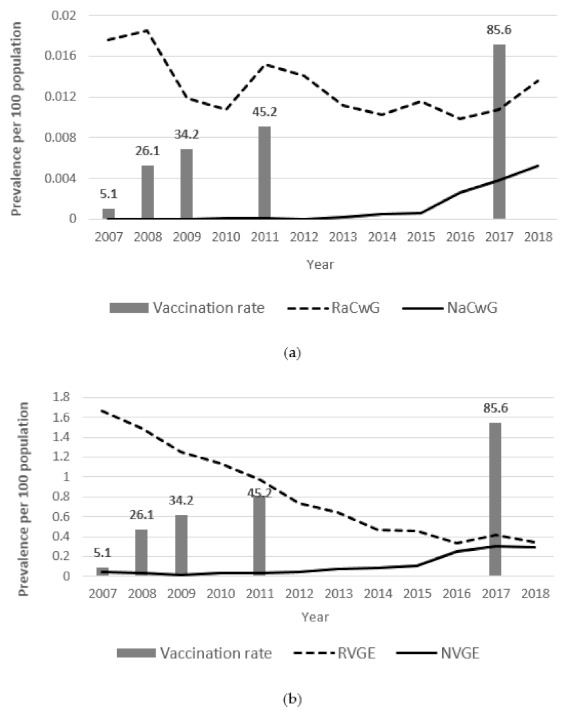
Annual changes of the prevalence of rotavirus gastroenteritis (RVGE), rotavirus-associated benign convulsion with mild gastroenteritis (RaCwG), norovirus gastroenteritis (NVGE) and norovirus gastroenteritis (NaCwG) and the vaccination rate. (**a**) Annual changes of the RaCwG prevalence per 100 comprising the mid-year point (MYP) (Exp(B): 0.956, *p* = 0.190) and NaCwG patients per 100 comprising the MYP (Exp(B): 1.790, *p* < 0.001). (**b**) Annual changes of the prevalence of RVGE (Exp(B): 0.869, *p* < 0.001) and NVGE (Exp(B): 1.224, *p* < 0.001) per 100 comprising the MYP.

Although the rotavirus inoculation rate increased every year, the prevalence of RaCwG did not show any statistically significant change, but the prevalence of NaCwG increased by 1.790 times each year (*p* < 0.001) (Table 2). Furthermore, in all years of the study period, the prevalence of RaCwG was higher than that of NaCwG (Figure 1).

The prevalence of RVGE decreased 0.869 times each year (*p* < 0.001). However, the prevalence of NVGE increased by 1.224 times each year (*p* < 0.001) (Table 3). The prevalence of RVGE was higher than that of NVGE in all years of the study period (Figure 1).

**Figure 2:**
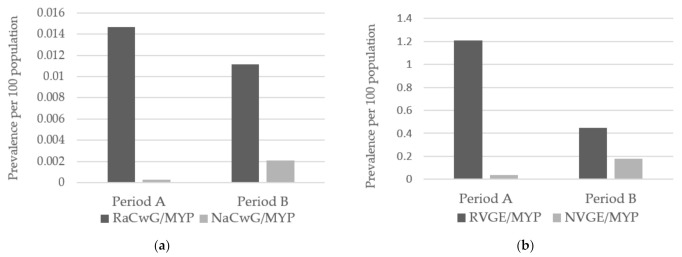
Between-period comparison of the prevalence of RVGE, RaCwG, NVGE and NaCwG. (**a**) Comparison of the RaCwG prevalence per 100 comprising the MYP (odds ratio: 0.759, *p* < 0.001) and NaCwG patients per 100 comprising the MYP (odds ratio: 115.311, *p* < 0.001). (**b**) Comparison of the prevalence of RVGE (odds ratio: 0.369, *p* < 0.001) and NVGE (odds ratio: 4.713, *p* < 0.001) per 100 comprising the MYP. RVGE: rotavirus gastroenteritis, RaCwG: rotavirus-associated benign convulsion with mild gastroenteritis, NVGE: norovirus gastroenteritis, NaCwG: norovirus-associated benign convulsion with mild gastroenteritis, MYP: mid-year population, Period A: 2007–2012, Period B: 2013–2018.

Regarding the comparison between periods A and B, the RVGE, RaCwG, NaCwG and NVGE prevalence per 100 of the MYP changed significantly from 1.20 to 0.45, 0.015 to 0.01, 0.000018 to 0.002 and 0.04 to 0.18, respectively, (*p* < 0.001) (Figure 2).

### 3.3. Annual Changes of the Ratio of CwG to RVGE or NVGE

**Figure 3:**
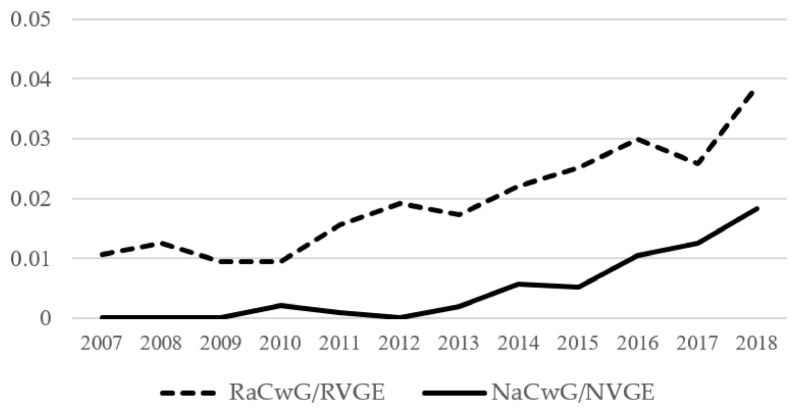
Annual changes in ratio of RaCwG to RVGE (Exp(β): 1.104 (95% CI 1.032–1.182), *p* = 0.004) and ratio of NaCwG to NVGE (Exp(β): 1.578 (95% CI 1.279–1.948), *p* < 0.001). RVGE: rotavirus gastroenteritis, RaCwG: rotavirus-associated benign convulsion with mild gastroenteritis, NVGE: norovirus gastroenteritis, NaCwG: norovirus-associated benign convulsion with mild gastroenteritis.

There was an annual increase in the ratio of RaCwG to RVGE by 1.104 (*p* = 0.004). The ratio of NaCwG to NVGE showed a significant annual increase by 1.852 (*p* < 0.001) (Table 4). In all years of the study period, the ratio of RaCwG to RVGE was higher than the ratio of NaCwG to NVGE (Figure 3).

### 3.4. The Prevalence of RaCwG, RVGE, NaCwG, NVGE and the Ratio of CwG to RVGE or NVGE According to Season

There was no statistically significant difference in the prevalence of RaCwG and NaCwG according to the season (Table 2). The incidence of RVGE was significantly less during two seasons, with an odds ratio in summer of 0.201 (*p* = 0.049) and an odds ratio in autumn of 0.196 (*p* = 0.042), relative to spring. There was no significant difference in the prevalence of NVGE according to the season (Table 3).

The ratio of RaCwG to RVGE and the ratio of NaCwG to NVGE were not significantly different depending on the season (Table 4).

**Figure 4:**
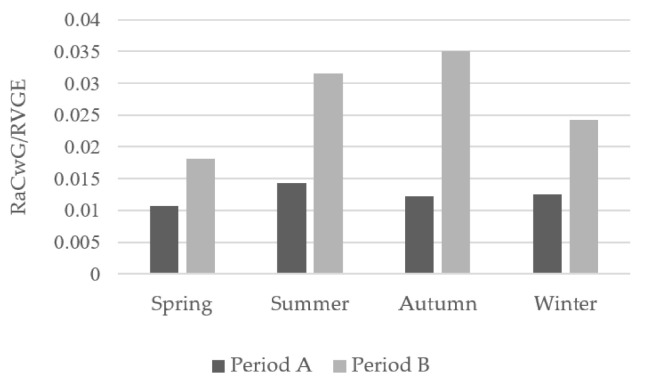
Seasonal between-period comparison of the ratio of RaCwG to RVGE, *p* = 0.298. RVGE: rotavirus gastroenteritis, RaCwG: rotavirus-associated benign convulsion with mild gastroenteritis, Period A: 2007–2012, Period B: 2013–2018.

The ratio of RaCwG to RVGE was highest in the summer and autumn in periods A and B (Figure 4). The ratio was not significantly different in all seasons for period A and period B (*p* = 0.298).

### 3.5. The Prevalence of RaCwG, RVGE, NaCwG, NVGE and the Ratio of CwG to RVGE or NVGE According to Sex

The prevalence of RaCwG was significantly lower in females than in males and the odds ratio was 0.921 (*p* = 0.007). However, the prevalence of NaCwG did not differ significantly according to sex (Table 2). The prevalence of RVGE was significantly lower in females than in males and the odds ratio was 0.846 (*p* = 0.004). However, the prevalence of NVGE did not show any significant difference according to sex (Table 3).

The ratio of RaCwG to RVGE and the ratio of NaCwG to NVGE were not significantly different according to sex (Table 4).

### 3.6. The Prevalence of RaCwG, RVGE, NaCwG, NVGE and the Ratio of CwG to RVGE or NVGE According to Age

The prevalence of RaCwG was decreased in all age groups from 1 to 6 years old compared to those under 1 year old (*p* < 0.001). Moreover, based on the age group under 1 year old, the odds ratio decreased as the age increased (Table 2).

The prevalence of NaCwG was significantly higher in the age group between 1 and 2 years old compared to the age group under 1 year old (*p* < 0.001). However, the prevalence was significantly lower in the age group from 3 to 6 years old compared to the age group under 1 year (*p* < 0.001) (Table 2).

The prevalence of RVGE was significantly lower in all age groups between 2 and 6 years of age compared to those under 1 year of age. As the age increased, the odds ratio for those under 1 year old decreased (Table 3).

The prevalence of NVGE was significantly higher in the age group between 1 and 2 years old compared to the age group under 1 year old (*p* < 0.001). However, the prevalence was significantly lower in the age group from 2 to 6 years old compared to the age group under 1 year (*p* < 0.001) (Table 2).

The ratio of RaCwG to RVGE was significantly lower in all age groups from 2 to 6 years old compared to the age group under 1 year. At this time, the odds ratio was lower as the age group increased. The ratio of NaCwG to NVGE was significantly lower in all age groups from 5 to 6 years old compared to the age group under 1 year. However, the odds ratio in the age group from 1 to 2 years was higher than that of the age group under 1 year (Table 4).

## 4. Discussion

Most patients with CwG do not present with abnormal findings in the cerebrospinal fluid test, blood test and electroencephalogram. Further, there are no convulsions after improvement of digestive symptoms; additionally, anticonvulsants are rarely required since patients show a good long-term prognosis [23]. Therefore, further tests can be avoided through early CwG diagnosis. Numerous CwG cases are accompanied by rotavirus infection [10,24]. However, the incidence of norovirus, which has recently increased, is higher during winter than other seasons, which consequently affects the NaCwG prevalence.

In Korea, RotaTeq® was first introduced in 2007; however, since vaccination was not mandatory, the coverage increased after several years. In our study, the RVGE prevalence decreased after the introduction of the rotavirus vaccination, which is consistent with previous reports of a post-vaccination decrease in the prevalence of rotavirus enteritis [19,25,26].

In this study, vaccination affected the decrease in the RVGE prevalence. In addition, as the rotavirus vaccination rate increased, the prevalence of RaCwG decreased. However, the prevalence of RaCwG has not been declining every year. A recent study on patients with RaCwG aged <3 years reported a non-significant change in the RaCwG prevalence after rotavirus vaccination [27]. However, another study on patients aged <5 years reported a decrease in the hospitalization rate for convulsions after rotavirus vaccination [28]. In addition, there are studies showing that rotavirus vaccination reduces hospitalization rates for febrile seizures by 38%. This is a case that does not meet the diagnostic criteria for CwG, which is characterized by afebrile convulsion [29], but is a study showing that the rotavirus vaccine reduces the severity of neurological symptoms caused by rotavirus infection. From these results, the prevalence of RaCwG was influenced by other factors such as the age of the patient group as well as the rotavirus vaccination rate.

In 2006, a new norovirus variant termed GII.4 variant spread through groundwater and infected 2000 students in Korea [30]. Other variants were reported in 2009 and 2012, which led to large-scale diagnostic testing for norovirus [31]. In this study, there was an increase in the prevalence of NVGE and NaCwG after the introduction of the rotavirus vaccine. Moreover, this appears to reflect the recent trend of an increased norovirus prevalence in Korea [20,32]. Additionally, it is consistent with previous reports of an increase in the NVGE prevalence in Asia, Europe and the United States [33,34,35]. In Korea, norovirus enteritis was classified as a nationally designated infectious disease in 2011 and specimen surveillance began. Therefore, this may increase the availability of Norovirus diagnostic tests, which may affect the prevalence of NVGE and NaCwG. Wwith these changes, RT-PCR began to be used for the diagnosis of norovirus [36]. RT-PCR tends to have higher sensitivity than ELISA used in the past and this can also be a factor that increases the prevalence of NVGE and NaCwG [37,38].

We found that RVGE, RaCwG, NVGE and NaCwG showed a higher prevalence in spring and winter, which is consistent with previous findings [13,17,39]. There was no significant change in the seasonal ratio of RaCwG to RVGE after rotavirus vaccination, which suggests that the rotavirus vaccination helped lower the RaCwG prevalence, but not the ratio of RaCwG to RVGE.

In this study, patients with RVGE and RaCwG had the highest proportions among the <1 year group, which is consistent with previous findings [40,41]. Moreover, patients with NVGE and NaCwG had the highest proportions among children aged 1–2, which is consistent with the findings of previous single-center studies [42,43]. In all four diseases, the prevalence of patients decreased with age in the age group of 3 to 6 years old, and the ratio of RaCwG to RVGE also showed a decreasing trend in the same age group. This is thought to be due to the neurological vulnerability of children in younger age groups [44].

We observed a significant decrease in the RaCwG prevalence after rotavirus vaccination; however, the ratio of RaCwG to RVGE increased. Therefore, other factors could have contributed to the increased RaCwG ratio. First of all, rotavirus vaccination not only lowers the prevalence of RVGE, but also reduces the severity of RVGE, which can reduce the frequency of patient visits to the hospital [45,46]. Besides, it was recently reported that urban areas have a higher RaCwG prevalence than rural areas [27]. According to the KOSIS data, there has been a gradual increase in the size of the urban population in South Korea. Therefore, factors associated with the residence of the patients may affect the ratio of RaCwG to RVGE and NaCwG to NVGE. Furthermore, uric acid can be used as a diagnostic index for RaCwG; therefore, the effects of uric acid on the ratio of RaCwG to RVGE should be considered [47]. The ratio of NaCwG to NVGE consistently increased regardless of rotavirus vaccination. Thus, since there was an increasing trend in the NVGE prevalence, factors other than the increased number of patients with NVGE contributed to the increased NaCwG prevalence. There are reports that a family history is associated with the occurrence of CwG, suggesting that genetic factors may influence the prevalence of CwG [48,49]. For example, there are several studies on the effect of mutations in the neuronal sodium channel alpha 1 subunit (SCN1A) gene on the pathogenesis of CwG [50,51,52]. During the study period, there was a continuous increasing trend in the ratio of RaCwG to RVGE; moreover, the ratio of NaCwG to NVGE showed a similar increase in the non-vaccinated norovirus group. Therefore, it can be considered that vaccination against the enteritis virus does not improve the ratio of CwG to acute gastroenteritis.

This study has several limitations. First, we could not directly verify the participants’ medical records. Specifically, use of the HIRA database alone could not yield specific clinical information regarding the patients, including the type and duration of convulsion; and it was not possible to know exactly how the patient was diagnosed with rotavirus, norovirus enteritis or CwG associated with this virus. Second, diagnostic codes could be used to differentiate patients. In cases where diagnostic codes were omitted for patients with test results indicative of RVGE or NVGE, the number of patients would have been as low as those excluded from the study. Third, this study did not sufficiently consider the coinfection of rotavirus and norovirus due to technical limitations in the process of using the HIRA database. Because of this, the prevalence of each disease can be calculated higher than it actually is.

Our findings revealed a decrease in the prevalence of RVGE and RaCwG after rotavirus vaccination. However, NVGE and NaCwG, which were not associated with rotavirus vaccination, showed a continuously increased prevalence. This suggests that rotavirus vaccination is effective for RVGE-caused gastrointestinal symptoms and CwG, which is related to the nervous system. Additionally, it may be helpful in differential diagnosis of seizures for clinicians to keep in mind the possibility of CwG in children under 2 years of age. There is also a need to develop a norovirus vaccine to reduce the incidence of NVGE and NaCwG. However, since the ratio of RaCwG to RVGE and NaCwG to NVGE continuously increased, further studies are required to identify other factors that increase the progression of CwG to acute gastroenteritis. Moreover, there is a need to determine whether the prevalence of CwG continues to increase overall.

## Figures and Tables

**Table 1:** Characteristics of patients with rotavirus gastroenteritis (RVGE), rotavirus-associated benign convulsion with mild gastroenteritis (RaCwG), norovirus gastroenteritis (NVGE) and norovirus-associated CwG (NaCwG).

	RVGE	RaCwG	NVGE	NaCwG
Subjects	273,898	4246	35,593	337
Sex				
Male	150,150 (54.8)	2211 (52.1)	19,439 (54.6)	153 (45.4)
Female	123,748 (45.2)	2035 (47.9)	16,154 (45.4)	184 (54.6)
**Age (years)**				
<1	103,616 (37.8)	2681 (63.1)	7905 (22.2)	47 (13.9)
1	76,690 (28.0)	974 (23.0)	10,638 (30.0)	202 (59.9)
2	39,279 (14.3)	376 (8.9)	5901 (16.6)	48 (14.2)
3	24,970 (9.1)	115 (2.7)	4215 (11.8)	14 (4.2)
4	17,122 (6.3)	60 (1.4)	3611 (10.1)	17 (5.0)
5	12,221 (4.5)	40 (0.9)	3323 (9.3)	9 (2.7)
Season				
Spring	94,912 (34.7)	1211 (28.5)	8508 (23.9)	72 (21.4)
Summer	43,350 (15.8)	833 (19.6)	4676 (13.1)	46 (13.6)
Autumn	44,694 (16.3)	805 (19.0)	6560 (18.4)	49 (14.5)
Winter	90,942 (33.2)	1397 (32.9)	15,849 (44.5)	170 (50.4)
Year				
2007	47,173 (17.2)	501 (11.8)	1420 (4.0)	0
2008	41,619 (15.2)	518 (12.2)	1107 (3.1)	0
2009	34,556 (12.6)	327 (7.7)	513 (1.4)	0
2010	31,232 (11.4)	293 (6.7)	937 (2.6)	2 (0.6)
2011	26,843 (9.8)	419 (9.8)	1059 (3.0)	1 (0.3)
2012	20,472 (7.5)	392 (9.2)	1377 (3.9)	0
2013	18,074 (6.6)	312 (7.3)	1978 (5.5)	4 (1.2)
2014	12,778 (4.7)	282 (6.6)	2257 (6.3)	13 (3.9)
2015	12,614 (4.6)	317 (7.5)	3047 (8.6)	16 (4.7)
2016	8949 (3.2)	267 (6.3)	6767 (19.0)	71 (21.0)
2017	10,907 (4.0)	282 (6.6)	7954 (22.3)	99 (29.4)
2018	8681 (3.1)	337 (7.9)	7177 (20.2)	131 (38.9)

RVGE: rotavirus gastroenteritis, RaCwG: rotavirus-associated benign convulsion with mild gastroenteritis, NVGE: Norovirus gastroenteritis, NaCwG: norovirus-associated benign convulsion with mild gastroenteritis. Values are presented as the number of patients (%). Bold to make it easier to distinguish variables.

**Table 2:** Negative binomial regression analysis of patients with RaCwG, patients with NaCwG and the mid-year population (MYP).

	RaCwG/MYP			NaCwG/MYP		
	Exp (B)	95% CI	*p* Value	Exp (B)	95% CI	*p* Value
**Age**						
<1	1	Reference		1	Reference	
1~2	343	0.319–0.369	<0.001 *	3.802	2.768–5.223	<0.001 *
2~3	0.131	0.117–0.146	<0.001 *	0.850	0.569–1.271	0.429
3~4	0.04	0.033–0.048	<0.001 *	0.242	0.133–0.440	<0.001 *
4~5	0.02	0.016–0.026	<0.001 *	0.289	0.166–0.504	<0.001 *
5~6	0.013	0.010–0.018	<0.001 *	0.148	0.073–0.303	<0.001 *
**Year**	0.956	0.894–1.022	0.19	1.790	1.683–1.904	<0.001 *
**Sex** (female)	0.921	0.867–0.978	0.007 *	1.539	0.521–4.546	0.435
**Season**						
Spring	1	Reference		1	Reference	
Summer	0.704	0.314–1.577	0.394	0.582	0.166–2.045	0.399
Fall	0.677	0.302–1.517	0.343	1.081	0.300–3.891	0.906
Winter	1.139	0.510–2.546	0.751	4.184	1.271–13.77	0.019 *

RaCwG/MYP: Number of patients with rotavirus-associated benign convulsions with mild gastroenteritis compared with the mid-year population. NaCwG/MYP: Number of patients with norovirus-associated benign convulsions with mild gastroenteritis compared with the mid-year population. CI: Confidence interval. * *p* < 0.05. Bold to make it easier to distinguish variables.

**Table 3:** Negative binomial regression analysis of patients with RVGE, patients with NVGE and the MYP.

	RVGE/MYP			NVGE/MYP		
	Exp(B)	95% CI	*p* Value	Exp(B)	95% CI	*p* Value
**Age**						
<1	1	Reference		1	Reference	
1~2	0.624	0.280–1.393	0.250	1.227	1.192–1.264	<0.001 *
2~3	0.339	0.152–0.755	0.008 *	0.656	0.635–0.679	<0.001 *
3~4	0.219	0.098–0.487	<0.001 *	0.461	0.444–0.478	<0.001 *
4~5	0.156	0.070–0.348	<0.001 *	0.39	0.375–0.406	<0.001 *
5~6	0.113	0.051–0.251	<0.001 *	0.353	0.339–0.367	<0.001 *
**Year**	0.869	0.814–0.938	<0.001 *	1.224	1.176–1.273	<0.001 *
**Sex** (female)	0.846	0.755–0.949	0.004 *	0.833	0.374–1.855	0.654
**Season**						
Spring	1	Reference		1	Reference	
Summer	0.201	0.201–0.995	0.049 *	0.568	0.255–1.266	0.167
Fall	0.196	0.196–0.972	0.042 *	0.73	0.327–1.628	0.442
Winter	0.402	0.402–1.992	0.784	1.759	0.789–3.922	0.167

RVGE/MYP: Number of patients with rotavirus gastroenteritis compared with the mid-year population. NVGE/MYP: Number of patients with norovirus gastroenteritis compared with the mid-year population. CI: Confidence interval. * *p* < 0.05. Bold to make it easier to distinguish variables.

**Table 4:** Negative binomial regression analysis of the patients with RaCwG compared with RVGE; the patients with NaCwG compared with NVGE.

	RaCwG/RVGE			NaCwG/NVGE		
	Exp(B)	95% CI	*p* Value	Exp(B)	95% CI	*p* Value
**Age**						
<1	1	Reference		1	Reference	
1~2	0.494	0.220–1.106	0.086	4.49	1.362–14.798	0.014 *
2~3	0.365	0.163–0.821	0.015 *	0.927	0.259–3.315	0.908
3~4	0.171	0.075–0.391	<0.001 *	0.286	0.070–1.171	0.082
4~5	0.133	0.057–0.309	<0.001 *	0.255	0.063–1.033	0.056
5~6	0.119	0.050–0.282	<0.001 *	0.140	0.031–0.630	0.010 *
**Year**	1.104	1.032–1.106	0.004 *	2.128	1.768–2.560	<0.001 *
**Sex** (female)	1.08	0.484–2.413	0.851	1.852	0.627–5.471	0.265
**Season**						
Spring	1	Reference		1	Reference	
Summer	1.542	0.688–3.457	0.293	1.086	0.307–3.838	0.899
Fall	1.539	0.685–3.459	0.297	1.384	0.385–4.980	0.619
Winter	1.263	0.565–2.822	0.569	2.279	0.684–7.596	0.180

RaCwG/RVGE: Number of patients with rotavirus-associated benign convulsions with mild gastroenteritis compared with rotavirus gastroenteritis. NaCwG/NVGE: Number of patients with norovirus-associated benign convulsions with mild gastroenteritis compared with norovirus gastroenteritis. CI: confidence interval. * *p* < 0.05. Bold to make it easier to distinguish variables.

## Data Availability

The data are not publicly available.
